# Familiarity and Novelty in Aesthetic Preference: The Effects of the Properties of the Artwork and the Beholder

**DOI:** 10.3389/fpsyg.2021.694927

**Published:** 2021-07-23

**Authors:** Jiwon Song, Yuna Kwak, Chai-Youn Kim

**Affiliations:** School of Psychology, Korea University, Seoul, South Korea

**Keywords:** preference, paintings, familiarity, novelty, content, visual complexity, art experience

## Abstract

Familiarity and novelty are fundamental yet competing factors influencing aesthetic preference. However, whether people prefer familiar paintings or novel paintings has not been clear. Using both behavioral and eye-tracking measures, the present study aimed to investigate whether the effect of familiarity-novelty on aesthetic preference is independent or dependent on artwork properties (painting content, visual complexity) and viewer characteristics (experience in art). Participants were presented with two images of paintings, one of which was repeatedly presented but was always paired with a new painting in a randomized lateral arrangement. They were asked to indicate which of the two images they preferred with the degree of their preference. Behavioral results demonstrated an interactive influence of painting content and complexity on familiarity-novelty preference, especially alongside the distinction between representational and abstract paintings. Also, the familiarity-novelty preference was modulated by the degree of art experience, for abstract paintings in particular. Gaze results showed the differential effects of painting content, complexity, and art experience echoing the behavioral results. Taken together, the convergent results derived from behavioral and eye-tracking measures imply that novelty is an important feature of aesthetic appreciation, but its influence is modulated by properties of both the artwork and the beholder.

## Introduction

“*An active striving to encounter new experiences, and to assimilate and understand them when encountered, underlies a huge variety of activities highly esteemed by society, from those of the scientist, the artist and the philosopher to those of the polar explorer and the connoisseur of wines*” (Berlyne, 1950).

Novelty seeking is not only one of the fundamental propensities of human beings (Fantz, 1964) but also a critical value that is pursued in art (Martindale, 1990; Hekkert and van Wieringen, 1996). Novelty is essential to understanding aesthetic preferences in the field of empirical aesthetics (Berlyne, 1950, 1970; Menninghaus et al., 2019), along with various perceptual (e.g., complexity, symmetry, golden ratio), emotional (e.g., pleasure, empathy, awe), and cognitive (e.g., knowledge, expertise, context) attributes that influence preference decisions (Imamoglu, 2000; Leder et al., 2004; Chatterjee and Vartanian, 2014; Chassy et al., 2015; Street et al., 2016; Jacobsen and Beudt, 2017; Tiihonen et al., 2017).

Novelty and familiarity, which are two sides of the same coin that influences aesthetic preference, have been recognized as important components of aesthetic appreciation at a relatively early processing stage subsequent to perceptual analyses (Leder et al., 2004; Pelowski et al., 2017) and as competing components about aesthetic preference (Bornstein, 1989; Leder and Nadal, 2014; Montoya et al., 2017). As one of prominent theories of aesthetic preference, the processing fluency theory proposed that the more fluently perceivers can process objects, the more positively they appreciate them (Reber et al., 2004; Reber, 2012). One of the determinants of fluency is a prior exposure, as evidenced by the mere exposure effect suggesting a higher preference for frequently exposed, familiar stimuli (Zajonc, 1968; Bornstein, 1989; Cutting, 2007). The fluency theory, however, conflicts with striving for novelty and innovativeness which is a dominant force in the development of art (Martindale, 1988; Arnason and Mansfield, 2013). A number of studies have demonstrated that people highly appreciated novel, challenging, and ambiguous stimuli (Jakesch and Leder, 2009; van de Cruys and Wagemans, 2011; Muth and Carbon, 2013, 2016; Muth et al., 2013, 2015; Belke et al., 2015) which has been buttressed by a neural substrate of the pleasure of acquiring novel visual information (Biederman and Vessel, 2006; Wittmann et al., 2007). In addition, a third line of research suggested that the moderate degree of novelty, neither familiarity nor novelty, was preferred the most (Berlyne, 1974; Hekkert et al., 2003; Giora et al., 2004; Hekkert, 2006). Yet, it has not been clarified in the literature whether a novel artwork is always preferred to a familiar one or how novelty interacts with other factors affecting aesthetic preferences.

To operationally define and quantitatively measure novelty and familiarity, researchers have manipulated familiarity more often than novelty (Zajonc, 1968; Bornstein, 1989; Monin, 2003; Leder et al., 2004; Cela-Conde et al., 2011; Bohrn et al., 2013). Familiarity has been primarily defined in two different ways. Some studies have measured differences in observers' initial levels of familiarity with artworks (Leder, 2001; Cutting, 2003). For instance, familiarity has been quantified as the frequency of appearance of images of paintings in the books of a certain library and on- and off-line searches (Cutting, 2003). Familiarity has also been defined in terms of subjective ratings that participants provide to judge reproductions of van Gogh's paintings (Leder, 2001). Overall, this line of studies has shown that familiarity is correlated positively with preference. In contrast, other studies have experimentally manipulated familiarity through repetitive presentations (Berlyne, 1970; Imamoglu, 1974; Stang, 1974; Stang and O'Connell, 1974; Oskamp and Scalpone, 1975; Kruglanski et al., 1996) by using the “mere-exposure paradigm” (Zajonc, 1968). However, the studies have yielded inconsistent results: repeated exposure to an artwork leads to a corresponding increase in preference (Kruglanski et al., 1996) whereas repeated exposure to an abstract pattern decreased aesthetic preference for the respective pattern (Imamoglu, 1974; Stang, 1974). Taken together, the relationship between familiarity-novelty and aesthetic preference remains unclear.

It is noteworthy that stimulus content, the representational-abstract dimension of the paintings in particular, is an important factor in modulating the effect of familiarity-novelty on aesthetic preference and, therefore, a potential reason behind the inconsistency. Previous studies have shown that aesthetic preference differs depending on whether the depicted contents in the paintings are recognizable or not, namely, representational and abstract paintings (Knapp and Wulff, 1963; Kettlewell et al., 1990; Furnham and Walker, 2001; Fairhall and Ishai, 2008; Vessel and Rubin, 2010; Pihko et al., 2011; Muth and Carbon, 2013; Pelowski et al., 2017), and that appreciation of them involves different neural correlates (Kettlewell and Lipscomb, 1992; Lengger et al., 2007; Cattaneo et al., 2014a,b; Cattaneo et al., 2015, 2017). Furthermore, representational paintings tend to be appreciated based on the contents that were portrayed in the paintings, rather than artwork *per se* or artistic style (Augustin et al., 2008, 2011; Leder et al., 2013). Considering these distinctions between representational and abstract paintings in aesthetic appreciation and preference, it is plausible that familiarity-novelty preference varies according to painting contents. Nevertheless, most of previous studies have examined the familiarity-novelty preference using a particular type of paintings such as portraits (Berlyne, 1970) or abstract paintings (Zajonc et al., 1972; Kruglanski et al., 1996). In the present study, we classified paintings into representational and abstract paintings and subdivided representational paintings into portraits and landscapes. We sought to investigate whether the effect of familiarity-novelty on aesthetic preference differs across the three painting contents (i.e., portraits, landscapes, and abstract paintings).

Visual complexity is considered as another mediating factor of familiarity-novelty preference. In a classic study of Berlyne (1970), it was specifically proposed that as paintings become familiar, simple paintings tend to be perceived as less pleasant, whereas complex paintings tend to be perceived as more pleasant. Since Berlyne's initial proposal on the role of complexity in aesthetic preference along the dimension of familiarity-novelty, studies have included complexity as a factor in the mere-exposure paradigm by classifying abstract images into two categories: simple and complex. However, evidence supporting the interaction between complexity and familiarity-novelty preference has been limited; irrespective of complexity, observers tend to prefer novel images (Stang, 1974; Oskamp and Scalpone, 1975) or show a gradual increase and a subsequent decrease in preference as exposure frequency increases (Stang and O'Connell, 1974). Therefore, it is worthwhile to reexamine complexity when examining the effect of familiarity-novelty on aesthetic preference.

In addition to the properties of the artworks including painting content and complexity, the art-related experience of the viewer is closely related to aesthetic preference and appreciation (Leder et al., 2004). It has been shown that art experts and non-experts tend to diverge on the assessment of abstract artworks compared to representational ones (Pihko et al., 2011; Leder et al., 2014; Mullennix and Robinet, 2018). This may be attributed to the fact that artistically naïve observers mainly focus on recognizable elements to derive meaning whereas artistically experienced viewers are able to derive meaning from the sensory features and the art medium (Cupchik and Gebotys, 1988; Schepman and Rodway, 2021). In addition, the large individual difference of aesthetic preference and enjoyment of abstract art is related to interest, knowledge, and activities relevant to art (Cattaneo et al., 2014b). Therefore, it stands to reason that this study examines whether the familiarity-novelty preference is modulated by the degree of art experience, and whether the influence is distinctive between representational and abstract paintings.

In light of previous evidence demonstrating the content-specific segregation of familiarity and novelty preference (Imamoglu, 1974; Stang, 1974; Biederman and Vessel, 2006; Park et al., 2010; Liao et al., 2011) and the relationship between visual complexity and familiarity-novelty preference (Berlyne, 1970), we hypothesized that familiarity-novelty preference differs across paintings of varied contents (portraits, landscapes, abstract paintings), varied degree of complexity (simple, complex), and the interaction between the two. We also hypothesized that familiarity-novelty preference is modulated by the degree of art experience, especially along the representational-abstract dimension of paintings.

To test aforementioned hypotheses, we used a sequential preference-judgment task in which participants were presented with a pair of images of paintings and required to indicate which of the two images they preferred and to what extent. One of the two images was repeatedly presented but was always paired with a new painting (Park et al., 2010). Such design allowed us to experimentally manipulate familiarity-novelty through repetition. It also enabled us to make a direct comparison between familiar and novel paintings as two alternatives and to observe changes in relative preference over trials, which the mere-exposure paradigm does not allow.

In addition to the direct and subjective behavioral measures, we monitored participants' eye movements when they viewed images of paintings and made an aesthetic preference judgment. Eye-tracking is an effective tool to examine the relationship between gaze and aesthetic preference (Holmes and Zanker, 2012). It has been shown that people fixated on aesthetically preferred abstract patterns more often and for longer durations (Williams et al., 2018). Longer fixation duration has also been reported for preferred paintings of Mondrian's (Plumhoff and Schirillo, 2009). Taken together, results from these studies imply that gaze is a predictor of aesthetic preference, for abstract paintings in particular. In the present study, we utilized eye-tracking measures to investigate whether gaze is directed toward familiar or novel paintings and whether it echoes familiarity-novelty preference, as inferred from behavioral measures. By examining convergence between behavioral aesthetic judgments and gaze responses, we expected to draw concrete conclusions about familiarity-novelty preference.

## Preliminary Survey for Stimulus Selection

To select the images of paintings for the main experiment, we conducted a preliminary survey in which participants rated each painting on four aspects: familiarity, preference, complexity, and abstractness.

### Materials and Methods

#### Participants

Two hundred and twenty-seven participants (portraits: *n* = 72 [49 females], landscapes: *n* = 85 [55 females], abstract paintings: *n* = 70 [48 females]) were recruited from Korea University and compensated for their participation. They had received no formal training in art, nor did they hold a degree in an art-related major. All participants had normal or corrected-to-normal vision. Informed consent was obtained from all participants in accordance with the procedures that were approved by the Institutional Review Board of Korea University [1040548-KU-IRB-16-199-A-1(E-A-1)].

#### Stimuli

Each category of paintings based on their content (portraits, landscapes, abstract paintings) consisted of a set of 200 color images that were sourced from WikiArt. Paintings with luminance histograms that were skewed to the left or right extreme were not included, in accordance with what has been previously suggested (Cela-Conde et al., 2004). All portrait stimuli were images of Impressionism and Post-Impressionism artworks. We selected images that portrayed the figure's upper body, not the whole body, and whose backgrounds were not outdoor scenes. All of them were in a portrait format (i.e., the heights of all images were longer than their widths). All landscape stimuli were images of Impressionism and Post-Impressionism artworks. We only included images that did not feature human figures. Paintings of the same painter of portrait stimuli were included as much as possible so that artistic styles were comparable across the different painting contents. All of them were in a landscape format (i.e., the widths of all images were longer than their heights). All abstract painting stimuli were images of artworks that were representative of Cubism, Neoplasticism, and Abstract Expressionism in Modern Art. Images that contained recognizable objects were excluded. Some images were in a portrait format, and others were in a landscape format.

#### Procedures

To experimentally manipulate familiarity-novelty through repetitive presentation, we aimed to select paintings that were moderately familiar (i.e., neither too familiar nor too novel at the beginning of the experiment). Further, we intended to select paintings that elicit moderate levels of preference; a painting that elicits extreme preferences is likely to be judged based on its original preference, rather than repetition manipulation during the experiment. Moreover, a strongly preferred or non-preferred painting is likely to influence preference of the subsequent painting, leading familiarity-novelty manipulation through repetitive presentation less effective (Cogan et al., 2013; Kondo et al., 2013; Tousignant and Bodner, 2014; Pegors et al., 2015; Chang et al., 2017; Khaw and Freedberg, 2018; Kim et al., 2019). Initial preference should be equally neutral across painting content and complexity conditions to prevent it from mediating familiarity-novelty preference (Peeters, 1971; Brickman et al., 1972; Kanouse and Hanson, 1972; Kruglanski et al., 1996). The variance of familiarity and preference across those stimulus conditions was also intended to be equal to keep the effectiveness of familiarity-novelty manipulation homogeneous across painting content and complexity conditions. In addition to the research questions that pertained to familiarity and preference, we included a question on complexity and aimed to subdivide paintings based on subjective rating scores. Finally, a question on abstractness was formulated with the objective of excluding paintings with easily recognizable objects for abstract paintings.

We conducted an online survey using SurveyMonkey (Portland, OR, USA). There were three types of survey that corresponded to the three painting categories based on contents (i.e., portraits, landscapes, and abstract paintings). Each survey comprised 200 images of paintings. For each painting content, there were two surveys that differed in the order in which paintings were presented. Each participant responded to only one of the six variations of the survey.

The survey consisted of items that required participants to provide demographic information (i.e., gender, age) and questions about the 200 painting images. Participants rated each painting on the following four dimensions using a 7-point scale:

1. Preference: Please rate how much you like the painting (−3 = dislike very much, 0 = neutral, 3 = like very much)
2. Familiarity: Please rate how familiar you are with the painting (−3 = very unfamiliar, 0 = neutral, 3 = very familiar)
3. Complexity: Please rate how visually complex the painting looks (−3 = very simple, 0 = neither simple nor complex, 3 = very complex)
4. Abstractness: Please rate how abstract the expression is (−3 = very representational, 0 = neither representational nor abstract, 3 = very abstract).

#### Data Analyses

The ratings that participants had provided for each of the four questions were transformed into normalized scores (Z-scores). Next, mean Z-scores were used to sort images in a descending order. We selected painting images with midrange scores for both familiarity and preference. To select images that elicited consistent responses from participants, we excluded images with large standard deviations (*SD* > 1.10) and selected images with smaller standard deviations. We discarded abstract painting images with low abstractness scores (*Z* < −0.40) that portrayed recognizable objects. Next, the selected images were subdivided into two groups, namely, simple and complex, based on complexity scores.

### Results

The mean familiarity Z-scores of all the images of paintings ranged from −1.07 to 2.67 (*SD* = 0.38). The mean preference Z-scores of all the images of paintings ranged from −1.27 to 1.04 (*SD* = 0.37). We selected images of paintings with midrange mean familiarity (−0.60 < *Z* < 0.60) and preference scores (−0.45 < *Z* < 0.45). The ranges of mean Z-scores as well as mean raw scores for all and the selected images in each category of paintings based on content are listed in Table 1.

**Table 1:** The ranges of Z-scores (raw scores) for all and the selected images of paintings in each category based on painting content.

**All paintings**		**Portraits**	**Landscapes**	**Abstract paintings**
	Familiarity	−1.07 < *Z* < 2.67 (−1.82 < *X* < 2.23)	−0.92 < *Z* < 0.80 (−1.55 < *X* < 0.42)	−0.90 < *Z* < 1.25 (−1.72 < *X* < 1.21)
	Preference	−1.23 < Z <1.04 (−1.63 < *X* < 0.97)	−1.27 < *Z* < 0.85 (−1.48 < *X* < 1.14)	−0.97 < *Z* < 1.01 (−1.86 < *X* < 0.83)
**Selected paintings**		**Portraits**	**Landscapes**	**Abstract paintings**
	Familiarity	−0.60 < *Z* < 0.60 (−1.01 < *X* < −0.01)	−0.60 < *Z* < 0.60 (−1.17 < *X* < 0.19)	−0.60 < *Z* < 0.60 (−1.32 < *X* < 0.18)
	Preference	−0.40 < *Z* < 0.40 (−0.68 < *X* < 0.25)	−0.40 < *Z* < 0.40 (−0.43 < *X* < 0.57)	−0.45 < *Z* < 0.45 (−1.24 < *X* < 0.10)

The selected images of paintings were subdivided into simple and complex groups based on their complexity Z-scores. Mean complexity scores (both Z and raw scores) and standard deviations for each complexity condition are shown in Table 2. One-way analysis of variance (ANOVA) revealed that the mean complexity scores of the simple and complex groups of images were significantly different, *F*_(1, 286)_ = 490.52, *p* < 0.001, $\eta_{\text{p}}^{2}$ = 0.63. Further, those within each category of painting content also demonstrated statistically significant differences (see Table 2).

**Table 2:** The mean complexity Z-scores and SD (raw scores, SD) in the six conditions and statistically significant differences between simple and complex groups of images within each category of painting content.

	**Portraits**	**Landscapes**	**Abstract paintings**
Simple	−0.35, 0.16 (−0.60, 0.17)	−0.36, 0.28 (−0.51, 0.33)	−0.54, 0.39 (−0.82, 0.64)
Complex	0.22, 0.22 (0.03, 0.24)	0.33, 0.20 (0.32, 0.24)	0.40, 0.30 (0.69, 0.48)
Statistics based on one-way ANOVA	*F*_(1, 94)_ = 211.92, *P* < 0.001	*F*_(1, 94)_ = 198.67, *p* < 0.001	*F*_(1, 94)_ = 168.28, *p* < 0.001

When selecting images of paintings for the main experiment, we ensured that there was no statistically significant difference in familiarity and preference ratings across the three painting content and two complexity conditions. Mean familiarity, preference scores, and standard deviations for each of the six conditions are listed in Table 3. With regard to familiarity, neither painting content, *F*_(2, 282)_ = 1.41, *p* = 0.25, nor complexity, *F*_(1, 282)_ = 1.99, *p* = 0.16, had a significant main effect on familiarity rating scores. The interaction effect between these two factors was not significant either, *F*_(2, 282)_ = 1.54, *p* = 0.22. Similarly, with regard to preference, no statistically significant main effect emerged for either painting content, *F*_(2, 282)_ = 0.02, *p* = 0.98, or complexity, *F*_(1, 282)_ = 0.33, *p* = 0.56. The interaction effect between painting content and complexity was not significant either, *F*_(2, 282)_ = 0.22, *p* = 0.80. Levene's tests showed that the variances across the six conditions were equal for both familiarity, *F*_(5, 282)_ = 0.94, *p* = 0.46, and for preference, *F*_(5, 282)_ = 0.33, *p* = 0.89, respectively.

**Table 3:** The mean familiarity and preference Z-scores and SD (raw scores, SD) for the selected images of paintings in the six conditions.

	**Portraits**	**Landscapes**	**Abstract paintings**
**Familiarity**			
Simple	0.04, 0.21 (−0.50, 0.26)	0.02, 0.20 (−0.40, 0.25)	0.01, 0.24 (−0.47, 0.34)
Complex	−0.02, 0.19 (−0.58, 0.22)	0.04, 0.23 (−0.37, 0.29)	−0.06, 0.23 (−0.55, 0.33)
**Preference**			
Simple	0.02, 0.22 (−0.18, 0.26)	0.00, 0.20 (0.03, 0.25)	0.01, 0.21 (−0.53, 0.31)
Complex	−0.01, 0.20 (−0.21, 0.23)	0.00, 0.21 (0.06, 0.28)	−0.01, 0.23 (−0.58, 0.32)

Accordingly, 96 images of paintings were selected for each of the three painting content conditions—namely, portraits, landscapes, and abstract paintings, each of which was further subdivided into two complexity conditions. There were 48 images of paintings in each of the six conditions. The complete list of the artworks selected for the main experiment is in Supplementary Material.

## Main Experiment

We conducted a sequential preference judgment task in which participants were asked to indicate which of the two paintings they preferred and their degree of preference. A total of 288 images of paintings, which had been selected based on the results of the preliminary survey, were used.

### Materials and Methods

#### Participants

Twenty-one participants (10 females, 11 males; M_age_ = 24.14 years, *SD* = 2.76) were recruited from Korea University and compensated for their participation. They had received no formal training in art, nor did they hold a degree in any art-related major. All of them had normal or corrected-to-normal vision. Written informed consent was obtained from all the participants in accordance with the procedures that were approved by the Institutional Review Board of Korea University [1040548-KU-IRB-16-199-A-1(E-A-1)].

#### Stimuli

**Figure 1:**
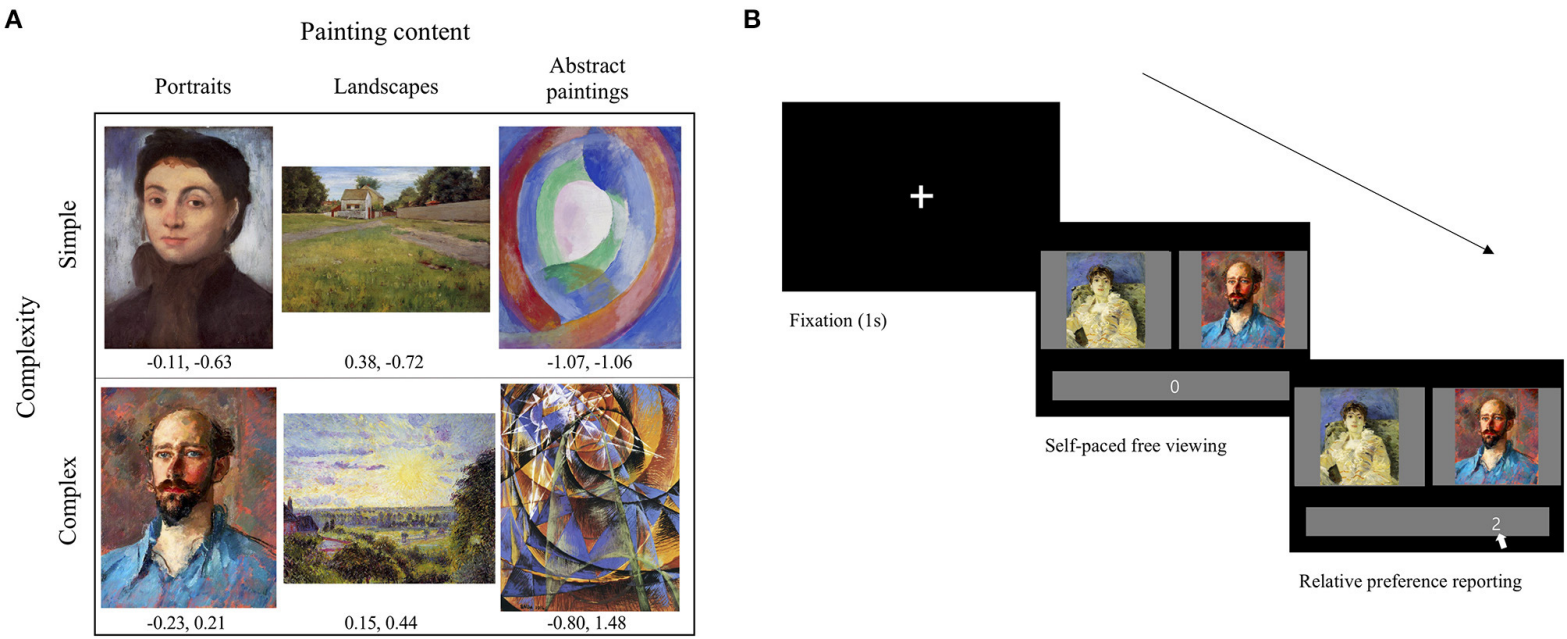
Stimuli and trial sequence. **(A)** Examples of images that were used in the experiments. All the paintings that are shown in this figure are copyright-free images that were sourced from WikiArt^1^. The values under the images are the mean raw scores of preference (left) and complexity (right) obtained from the preliminary survey. **(B)** An illustration of a sample experimental trial. After a fixation period of 1 s, two images of paintings were presented side by side, below which a response bar was presented. Participants were instructed to indicate which of the two paintings they preferred with their degree of preference on a 7-point scale that ranged from −3 to 3. The initial position of the mouse cursor was always on zero to avoid potential bias. There was no time limit for responses.

Based on the results of the preliminary survey, 96 images of paintings were selected for each of the three painting content conditions—namely, portraits, landscapes, and abstract paintings—and each of them was further subdivided into two complexity conditions. There were 48 images of paintings in each of the six conditions. Figure 1A depicts sample images that were used in each of the six conditions.

The size of each painting image was differentially adjusted based on their widths and heights. With regard to images of portraits, the height was specified as 13.10° of the visual angle, and the width was adjusted based on the aspect ratio, which remained unchanged. In the case of images of landscapes, the width was specified as 14.50° of the visual angle, and the height was adjusted accordingly. We presented a gray rectangle (32.13 cd/m^2^; 17.50° × 13.30°) behind each image of painting to minimize the influence of size variance of images.

#### Apparatus

Experiments were conducted in a quiet and dark room in which participants viewed a CRT monitor (1,024 × 768 resolution, 60-Hz frame rate, 55-cm distance), and heads were stabilized using a head and chin rest. The stimuli that were presented on the monitor were controlled using MATLAB version 9.1 (MathWorks, Inc., MA) and Psychophysics Toolbox Version 3 (Brainard, 1997; Pelli, 1997). Participants' left-eye movements were recorded at 500 Hz using a desk-mounted Eyelink 1000 Plus (SR Research, Mississauga, ON, Canada), which was controlled using Eyelink toolbox (Cornelissen et al., 2002) provided in the Psychophysics Toolbox. Calibration was undertaken using the conventional 5-point procedure, and eye position errors were found to be <0.5°.

#### Procedures

Subsequent to two practice trials, calibration for eye tracking was undertaken. Next, the main experiment was conducted. The experiment consisted of three blocks, each of which corresponded to each painting content. Each block consisted of six subblocks, three of which were simple conditions, and other three were complex conditions. The order of the blocks was counterbalanced across participants using a Latin square design, and the order of the subblocks was randomized. Each subblock consisted of 15 trials. Throughout each subblock of 15 trials, one of the two images was repeatedly presented but was always paired with a new painting in a randomized lateral arrangement (Park et al., 2010). The repeatedly presented (i.e., familiar) painting had the median level of preference within the subblock. This equalized the probabilities of preferring repeated (i.e., familiar) and novel paintings in each subblock. Participants completed a total of 272 trials (2 practice trials + 270 main trials [3 blocks × 6 subblocks × 15 trials]), which took about 30–40 min. A sample trial is illustrated in Figure 1B. Each trial began with a display of a white fixation cross on a black background for 1 s. Then, a pair of images from the same condition (i.e., out of the six conditions) was presented side by side, below which the response bar was presented. Participants were subjected to a sequential preference judgment task, whereby they were required to indicate which of the two images they preferred with their degree of preference on a 7-point scale that ranged from −3 to 3 (0 = neutral, −1 and 1 = slightly prefer, −2 and 2 = moderately prefer, −3 and 3 = strongly prefer). Irrespective of the response that participants had chosen in a previous trial, the initial position of the mouse cursor was always on 0 to avoid potential bias. There was no time limit to provide a response. Once a response had been provided, a blank screen was presented for 300 ms, following which the next trial was initiated.

After the completion of the main experiment, the experience in art of the participants was assessed using an Art Experience Questionnaire (Chatterjee et al., 2010). It is composed of eight questions that span three subscales: (1) education including classroom experience in studio art, art history, art theory, and aesthetics, (2) interest indicated by the frequency of visits to museums and galleries, and (3) activity indicated by the average time per week that is dedicated to art-related activities. Participants were required to rate their experiences using a 7-point Likert scale.

#### Data Analyses

##### Behavioral Analysis

**Figure 2:**
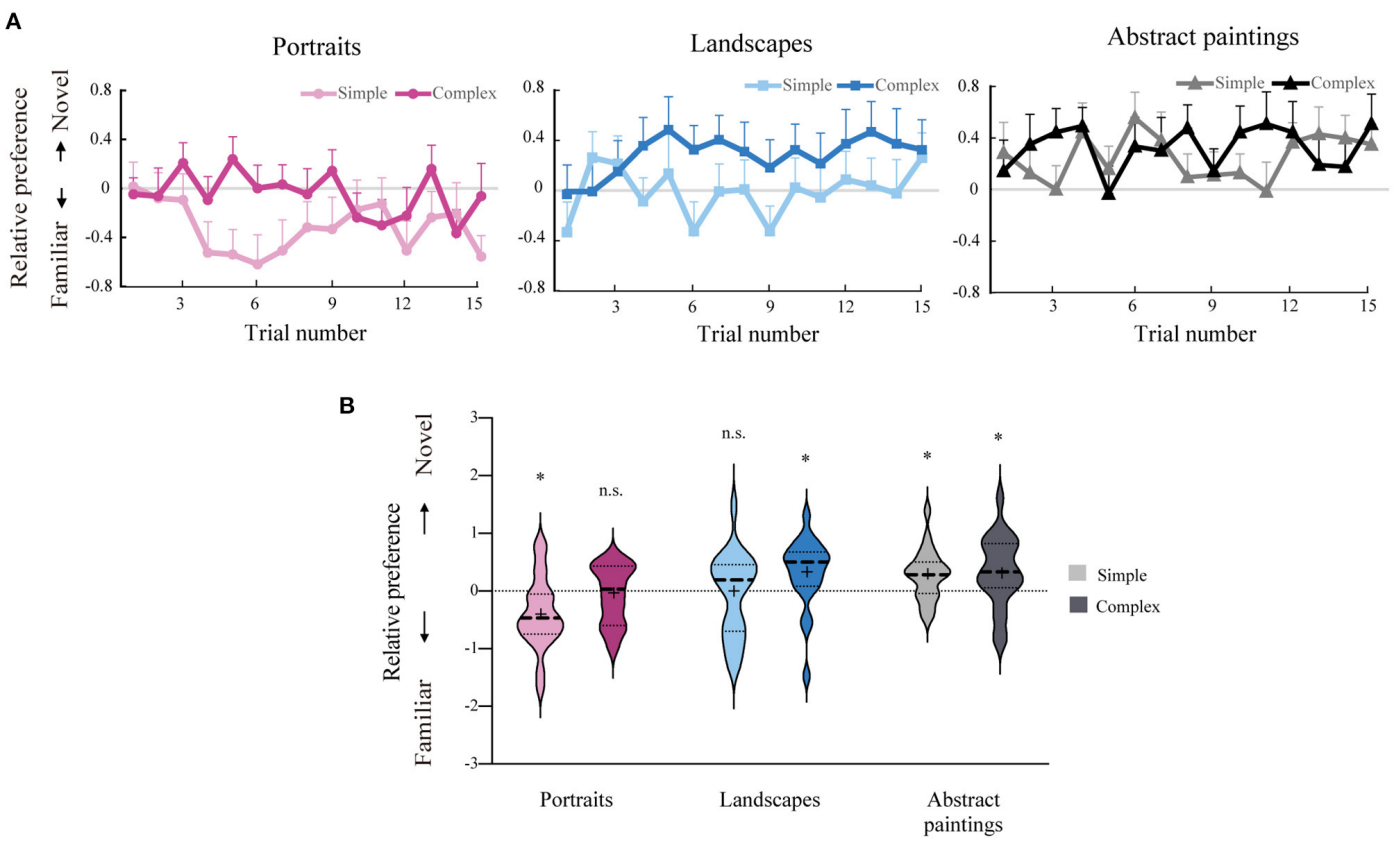
The time course and summary of familiarity-novelty preference. **(A)** The time course of familiarity-novelty preference. Time courses of preference values through a subblock were averaged for each complexity condition. The mean relative preference values are shown for each trial from the first to the fifteenth trial. The negative values of the y-axis indicate a preference for repeated (i.e., familiar) paintings, and the positive values of the y-axis indicate a preference for novel paintings. Error bars represent +1 standard error of the mean. **(B)** Summary of familiarity-novelty preference. Violin plots illustrate the distributions and the descriptive statistics of the individual mean relative preference values from the fourth to the fifteenth trial for each of the six conditions. The cross hairs indicate the mean. The middle horizontal lines indicate the median, and the lower and upper lines indicate the first and third quartiles. Asterisks indicate a significant difference from zero, as per the results of one-sample *t*-tests (*p < 0.05, n.s., not significant).

We calculated mean relative preference values for each trial across the three subblocks of each of the six conditions (Figure 2A). Negative values indicate a preference for repeated (i.e., familiar) paintings, whereas positive values indicate a preference for novel paintings. Another study that had used the paradigm that was adopted in the current study (Park et al., 2010) showed that participants became familiar with repeatedly presented images by the fourth trial. Our results also suggested the same trend; therefore, we included the values that were recorded between the fourth and fifteenth trials in the behavioral and gaze analyses.

In order to investigate whether the mean relative preference values of the 12 trials differ across the six conditions, we performed a two-way repeated measures ANOVA with two within-participant factors, namely, painting content (portraits, landscapes, abstract paintings) and complexity (simple, complex). To analyze the temporal changes in the relative preference value for each painting content, another two-way repeated measures ANOVA was performed with two within-participant factors of phase and complexity. Furthermore, correlation analyses were performed to assess the relationship between the mean relative preference values and the scores in art experience as a continuous independent variable, and between the mean relative preference values and each of the subscales, namely, education, interest, and activity.

##### Gaze Analysis

The eye tracking data of 18 out of 21 participants were collected. The data of two participants were not recorded as a result of technical problems with the eye-tracking system. The data of one participant were excluded due to a partial loss of the data. We focused on a gaze fixation analysis. Fixation duration threshold was specified as 100 ms (Salvucci and Goldberg, 2000), and fixations that were lower than this threshold were discarded. Fixations that were situated within the boundary of the gray rectangle (17.50° × 13.30°), which was presented behind each painting image, were analyzed.

We first calculated the average frequency with which participants had fixated upon familiar and novel paintings per trial, respectively. Then we computed a gaze index, defined as the ratio of fixation count for novel paintings relative to that for familiar paintings. The gaze index >1 indicates that participants' fixation was more frequently oriented toward novel paintings than toward repeated, familiar ones.

In order to examine whether the gaze index differs across the six conditions, we performed a two-way repeated measures ANOVA with painting content and complexity as the two within-participant factors. In addition, we conducted correlation analyses to assess the relationship between the gaze indices and art experience scores including each of the subscales of art education, interest, and activity.

### Results

#### Art Experience Questionnaire Results

The total scores of the Art Experience Questionnaire, and the scores for the three subscales are summarized in Table 4.

**Table 4:** The mean scores (SD) for the total and the three subscales of Art Experience Questionnaire.

	**Total**	**Education**	**Interest**	**Activity**
Mean (*SD*)	6.67 (4.75)	3.76 (2.90)	2.29 (1.95)	0.62 (0.80)

#### Behavioral Results

##### Familiarity-Novelty Preference in Terms of Painting Content and Complexity

Figure 2B shows the distributions and the mean of the individual mean relative preference values for each of the six conditions recorded between the fourth and fifteenth trials of the subblocks. A two-way repeated measures ANOVA with painting content (portraits, landscapes, abstract paintings) and complexity (simple, complex) as two within-participant factors revealed that painting content had a significant main effect on familiarity-novelty preference, *F*_(2, 40)_ = 8.02, *p* < 0.005, $\eta_{\text{p}}^{2}$ = 0.29. Complexity also had a significant main effect, *F*_(1, 20)_ = 10.80, *p* < 0.01, $\eta_{\text{p}}^{2}$ = 0.35. The interaction effect between painting content and complexity was not statistically significant, *F*_(1.42, 28.48)_ = 1.09, *p* = 0.33 (Greenhouse-Geisser corrected). *Post-hoc* pairwise comparisons of painting content conditions were conducted using paired *t*-tests. The false discovery rate (FDR)-correction was applied with *p* = 0.05 for multiple comparisons (Benjamini and Hochberg, 1995; Benjamini and Yekutieli, 2001). The degree of relative preference for familiar paintings was larger for portraits than for landscapes, *t*_(20)_ = 2.59, *p* < 0.05, Cohen's *d* = 0.70 (*FDR corrected*), and abstract paintings, *t*_(20)_ = 5.27, *p* < 0.01, Cohen's *d* = 1.22 (*FDR corrected*). These results indicate that familiarity-novelty preference for portraits was different from those for landscapes and abstract paintings. In addition, familiarity-novelty preference for complex paintings differed from that for simple paintings.

Additional paired *t*-tests were conducted to examine the effect of complexity in each condition of painting content. There was a significant difference in mean relative preference values between simple and complex portraits, *t*_(20)_ = 2.91, *p* < 0.05, Cohen's *d* = 0.57 (*FDR corrected*) and a marginally significant difference between simple and complex landscapes, *t*_(20)_ = 2.14, *p* = 0.067, Cohen's *d* = 0.55 (*FDR correcte*d). The difference in mean relative preference values between simple and complex abstract paintings was not significant, *t*_(20)_ = 0.28, *p* = 0.78 (*FDR corrected*). In order to examine the significance of familiarity-novelty preference in each of the six conditions, we employed one-sample *t*-tests (see Figure 2B for *p*-values with *FDR corrections for the six comparisons*). The results were indicative of participants' preference for familiar paintings in simple portraits and novel paintings that were complex landscapes. With regard to abstract paintings, novel paintings were preferred, irrespective of their complexity.

##### Familiarity-Novelty Preference and Art Experience

**Figure 3:**
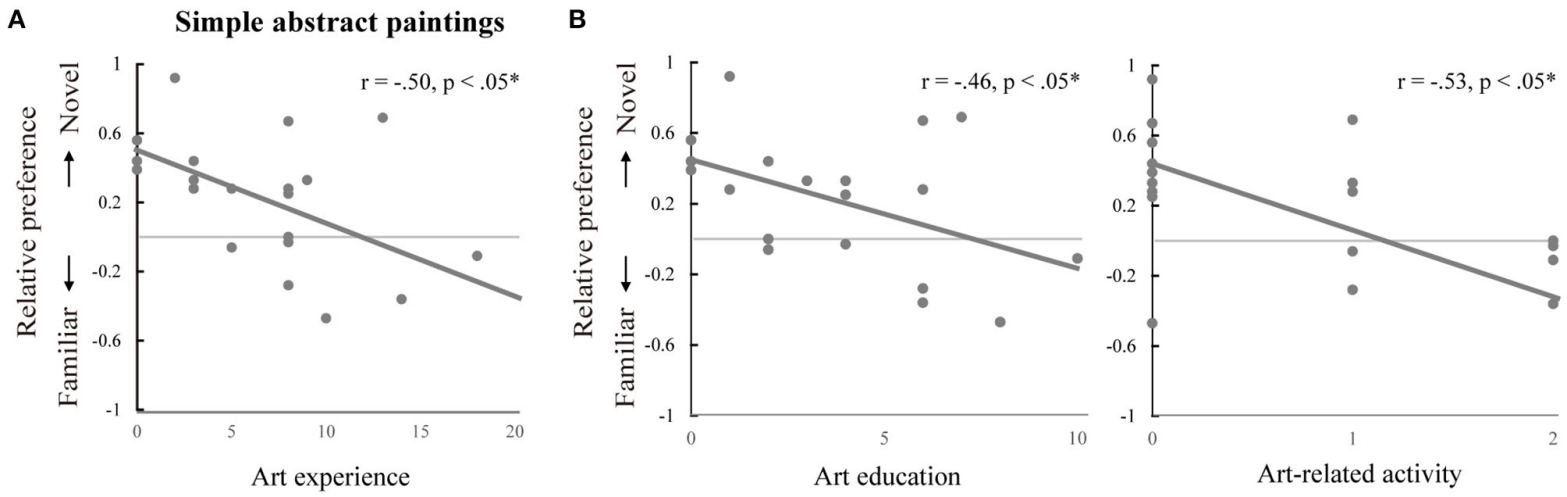
Familiarity-novelty preference and art experience in simple abstract paintings. **(A)** Correlation between the mean relative preference values and the total art experience scores. **(B)** Correlations between the mean relative preference values and the two subscale scores of art experience: education (left) and activity (right). *p < 0.05.

To examine whether familiarity-novelty preference was related to art experience, we performed an explorative correlation analyses between the mean relative preference values and the total art experience scores for each of the six conditions. The outlier (the mean relative preference value >2.5 *SD*) was excluded to satisfy an assumption about an absence of outliers. As shown in Figure 3A, there was a statistically significant negative correlation between the mean relative preference values for simple abstract paintings and the art experience scores, *r* = −0.50, *p* < 0.05. No statistically significant correlations were found for the other five conditions. Additional correlation analyses revealed that among the three subscales of art experience, education, *r* = 0.46, *p* < 0.05, and activity, *r* = −0.53, *p* < 0.05, were correlated negatively with familiarity-novelty preference for simple abstract paintings (Figure 3B).

Overall, these results suggested that participants who are less experienced in art prefer a novel painting to a familiar one in judging a pair of simple abstract paintings, which is highly associated with their education and activity components of art experience.

##### Temporal Changes of Familiarity-Novelty Preference

**Figure 4:**
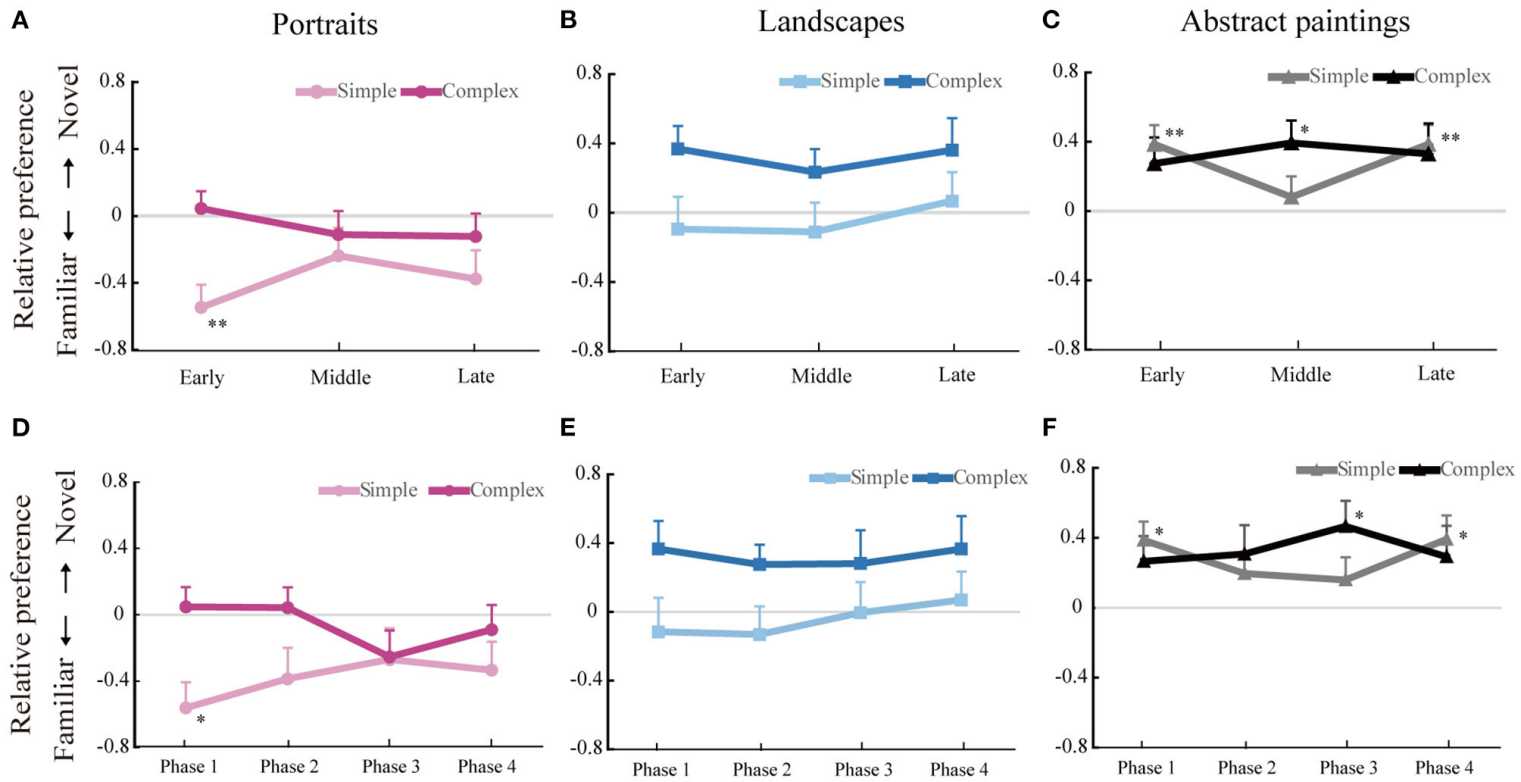
Temporal changes in familiarity-novelty preference. All trials between the fourth and fifteenth trials were divided into three phases: early (4–7), middle (8–11), and late (12–15) phases for portraits **(A)**, landscapes **(B)**, and abstract paintings **(C)**. All trials between the fourth and fifteenth trials were also divided into four phases: phase 1 (4–6), phase 2 (7–9), phase 3 (10–12), and phase 4 (13–15) phases for portraits **(D)**, landscapes **(E)**, and abstract paintings **(F)**. The mean relative preference values for each phase are shown. The negative values of the y-axis indicate a preference for repeated (i.e., familiar) paintings, and the positive values of the y-axis indicate a preference for novel paintings. Error bars represent +1 standard error of the mean. Asterisks indicate a significant difference from zero, as per the results of one-sample *t*-tests (*p < 0.05 and **p < 0.01).

As shown in Figure 2A, the familiarity-novelty preferences appear to fluctuate in the abstract paintings more than the representational paintings. To examine such apparent temporal fluctuation of preferences, the trials that spanned from the fourth to fifteenth trial were divided into three phases: early (4–7), middle (8–11), and late (12–15) phases within a subblock. This seemingly arbitrary division was chosen in consideration of the lack of a specific direction of changes over the whole trials. Temporal changes in each painting content are shown in Figure 4. An explorative two-way repeated measures ANOVA was conducted with phase and complexity as two within-participant factors. For portraits, complexity had a significant main effect on familiarity-novelty preference, *F*_(1, 20)_ = 8.47, *p* < 0.01, $\eta_{\text{p}}^{2}$ = 0.30 (Figure 4A). Neither the main effect of phase, *F*_(2, 40)_ = 0.52, *p* = 0.60, nor the interaction effect between the two factors, *F*_(2, 40)_ = 2.99, *p* = 0.06, was statistically significant. For landscapes, complexity had a significant main effect on familiarity-novelty preference, *F*_(1, 20)_ = 4.58, *p* < 0.05, $\eta_{\text{p}}^{2}$ = 0.19 (Figure 4B). Neither the main effect of phase, *F*_(2, 40)_ = 1.09, *p* = 0.35, nor the interaction effect between the two factors, *F*_(2, 40)_ = 0.57, *p* = 0.57, was statistically significant. For abstract paintings, the main effects of both phase, *F*_(1, 20)_ = 0.08, *p* = 0.78, and complexity, *F*_(2, 40)_ = 1.16, *p* = 0.32, on familiarity-novelty preference were not significant (Figure 4C). However, the interaction effect between the two factors was statistically significant, *F*_(2, 40)_ = 3.39, *p* = 0.04, $\eta_{\text{p}}^{2}$ = 0.15. A *post-hoc* analysis revealed that phase had a significant main effect on familiarity-novelty preference albeit only for simple abstract paintings, *F*_(2, 40)_ = 5.01, *p* = 0.01, $\eta_{\text{p}}^{2}$ = 0.20. When compared to the middle phase, novelty preference was more noticeable not only in the early phase, *t*_(20)_ = −2.96, *p* = 0.01, Cohen's *d* = 0.57 (*FDR corrected*), but also in the late phase, *t*_(20)_ = 3.14, *p* = 0.01, Cohen's *d* = 0.57 (*FDR corrected*), as per the results of *post-hoc* pairwise comparisons using paired *t*-tests. These results indicate that temporal changes in preference across the three phases were modulated by the complexity of abstract paintings, but this was not the case for portraits and landscapes.

In order to examine the significance of familiarity-novelty preference across the three phases in each of the six conditions, we employed one-sample *t*-tests (*with FDR corrections for the six comparisons*). As illustrated in Figures 4A,B, familiarity and novelty preferences were evident during the early phase for simple portraits, *t*_(20)_ = 4.00, *p* < 0.01, Cohen's *d* = 0.87 (*FDR corrected*), and complex landscapes, *t*_(20)_ = −2.79, *p* = 0.066, Cohen's *d* = 0.61 (*FDR corrected*), respectively. In Figure 4C, dynamic temporal changes in preferences for abstract paintings are shown. For complex abstract paintings, novelty preference was evident during the middle phase, *t*_(20)_ = −3.03, *p* < 0.05, Cohen's *d* = 0.66 (*FDR corrected*). For simple abstract paintings, novelty preference had emerged during the early, *t*_(20)_ = −3.45, *p* < 0.01, Cohen's *d* = 0.75 (*FDR corrected*), and late phases, *t*_(20)_ = −3.42, *p* < 0.01, Cohen's *d* = 0.75 (*FDR corrected*).

The results were consistent overall when the trials between the fourth and the fifteenth trial were divided into four, instead of three, phases: phase 1(4–6), phase 2 (7–9), phase 3 (10–12), and phase 4 (14–15) phases. As illustrated in Figures 4D,E, one-sample *t*-tests (*with FDR corrections for the four comparisons*) showed that familiarity preference was evident during the phase 1 for simple portraits, *t*_(20)_ = 3.64, *p* < 0.01, Cohen's *d* = 0.80 (*FDR corrected*), and during the phase 1 and 2 for complex landscapes, which was marginally significant, *t*_(20)_ = −2.22, *p* = 0.076, Cohen's *d* = 0.48 and *t*_(20)_ = −2.39, *p* = 0.076, Cohen's *d* = 0.52 (*FDR corrected*), respectively. In Figure 4F, dynamic temporal changes in preferences for abstract paintings are shown. For complex abstract paintings, novelty preference was evident during the phase 3, *t*_(20)_ = −3.18, *p* < 0.05, Cohen's *d* = 0.70 (*FDR corrected*). For simple abstract paintings, novelty preference had emerged during the phase 1, *t*_(20)_ = −3.63, *p* < 0.01, Cohen's *d* = 0.79 (*FDR corrected*), and the phase 4, *t*_(20)_ = −2.86, *p* < 0.05, Cohen's *d* = 0.63 (*FDR corrected*).

Taken together, for representational paintings, familiarity-novelty preference emerged early and tended to be maintained until the middle phase, whereas familiarity-novelty preference for abstract paintings underwent more dynamic changes as the trial progressed.

#### Gaze Results

##### The Ratio of Fixation Count for Novel to Familiar Paintings

**Figure 5:**
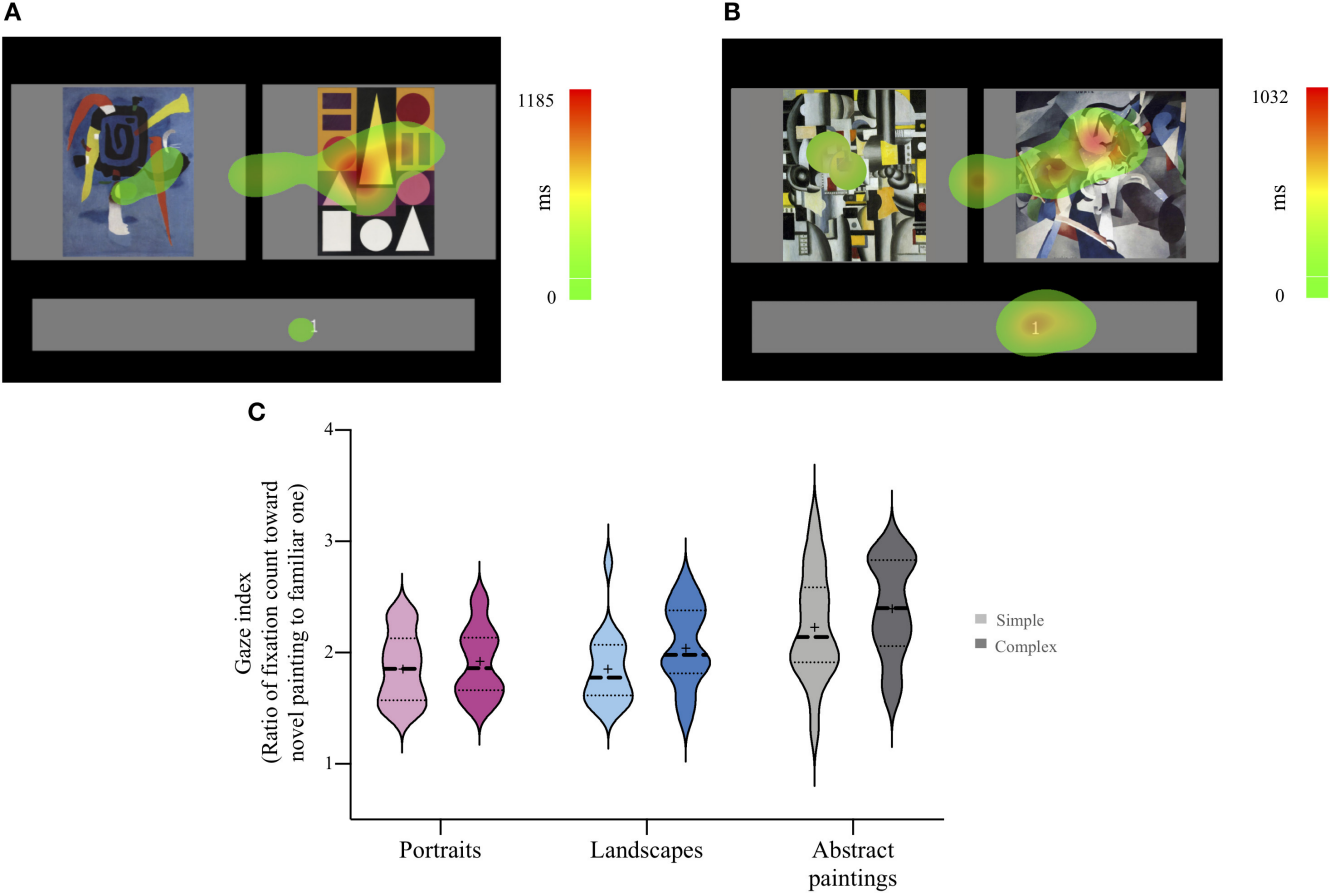
Fixation heat map and the ratio of fixation count. Fixation heat map for simple **(A)** and complex abstract painting **(B)** trials. A 2-dimensional Gaussian filter was applied to each of the fixations, and the height of the Gaussian was weighted by the duration of the individual fixations. **(C)** The gaze index (i.e., the ratio of fixation count for novel paintings to that for familiar paintings) was calculated. Violin plots illustrate the distributions and the descriptive statistics of the individual gaze index for each of the six conditions. The cross hairs indicate the mean. The middle horizontal lines indicate the median, and the lower and upper lines indicate the first and third quartiles. The larger a gaze index is than 1, the more frequently a participant had fixated on novel rather than repeated, familiar ones.

Figures 5A,B are heat maps that depict the fixation distribution for a sample trial with a simple and complex abstract painting, respectively. Repeated (i.e., familiar) and novel paintings are presented on the left and right sides of the figure, respectively. We calculated the average frequency with which participants had fixated upon familiar and novel paintings per trial. As exemplified in Figures 5A,B, most participants' fixation was more frequently oriented toward novel paintings across all six conditions. Therefore, our gaze index—i.e., the ratio of fixation count for novel paintings to that for familiar paintings—was >1 in most trials. The larger the difference between a gaze index value and 1 is, the more frequently the participant had fixated on novel rather than repeated, familiar ones.

The distributions and the means of individual gaze indices for each of the six conditions are shown in Figure 5C. A two-way repeated measures ANOVA with painting content and complexity as the two within-participant factors revealed that painting content had a significant main effect on the gaze index, *F*_(2, 34)_ = 15.82, *p* < 0.001, $\eta_{\text{p}}^{2}$ = 0.48. The main effect of complexity was also significant, *F*_(1, 17)_ = 9.29, *p* < 0.01, $\eta_{\text{p}}^{2}$ = 0.35. The results indicated that participants looked at novel paintings more frequently than familiar ones, when they viewed complex rather than simple paintings. The interaction effect between painting content and complexity was not statistically significant, *F*_(2, 34)_ = 0.78, *p* = 0.47. *Post-hoc* pairwise comparisons of painting content conditions using paired *t*-tests revealed that the gaze index for portraits was smaller than that for abstract paintings, *t*_(17)_ = −4.39, *p* < 0.001, Cohen's *d* = 1.22 (*FDR corrected*), and the gaze index for landscapes was smaller than that for abstract paintings, *t*_(17)_ = −4.31, *p* < 0.001, Cohen's *d* = 1.04 (*FDR corrected*). These results also indicate that participants looked at a novel painting more frequently than a repeated, familiar one when a pair of abstract paintings was presented than when a pair of representational paintings was presented. These results concur with the behavioral results, which are indicative of a general preference for novelty over familiarity for abstract paintings. Taken together, these results that emerged for the gaze index are comparable to the patterns of familiarity-novelty preferences that the behavioral results yielded.

It is noteworthy that, as mentioned under “Methods,” the gaze data of three out of 21 participants were not recorded or analyzed. Therefore, the behavioral and gaze results that have been reported in the preceding sections were derived using different numbers of participants (behavioral: n = 21, gaze: n = 18). To examine whether the convergence between the behavioral and gaze results may be attributable to these differences in samples, we reanalyzed the behavioral data of only those 18 participants whose data were included in gaze analysis. The results remained unchanged; the main effects of painting content, *F*_(2, 34)_ = 7.57, *p* < 0.01, $\eta_{\text{p}}^{2}$ = 0.31, and complexity, *F*_(1, 17)_ = 8.61, *p* < 0.01, $\eta_{\text{p}}^{2}$ = 0.34, were statistically significant even when the data of only the 18 participants were analyzed.

##### The Ratio of Fixation Count for Novel to Familiar Paintings and Art Experience

**Figure 6:**
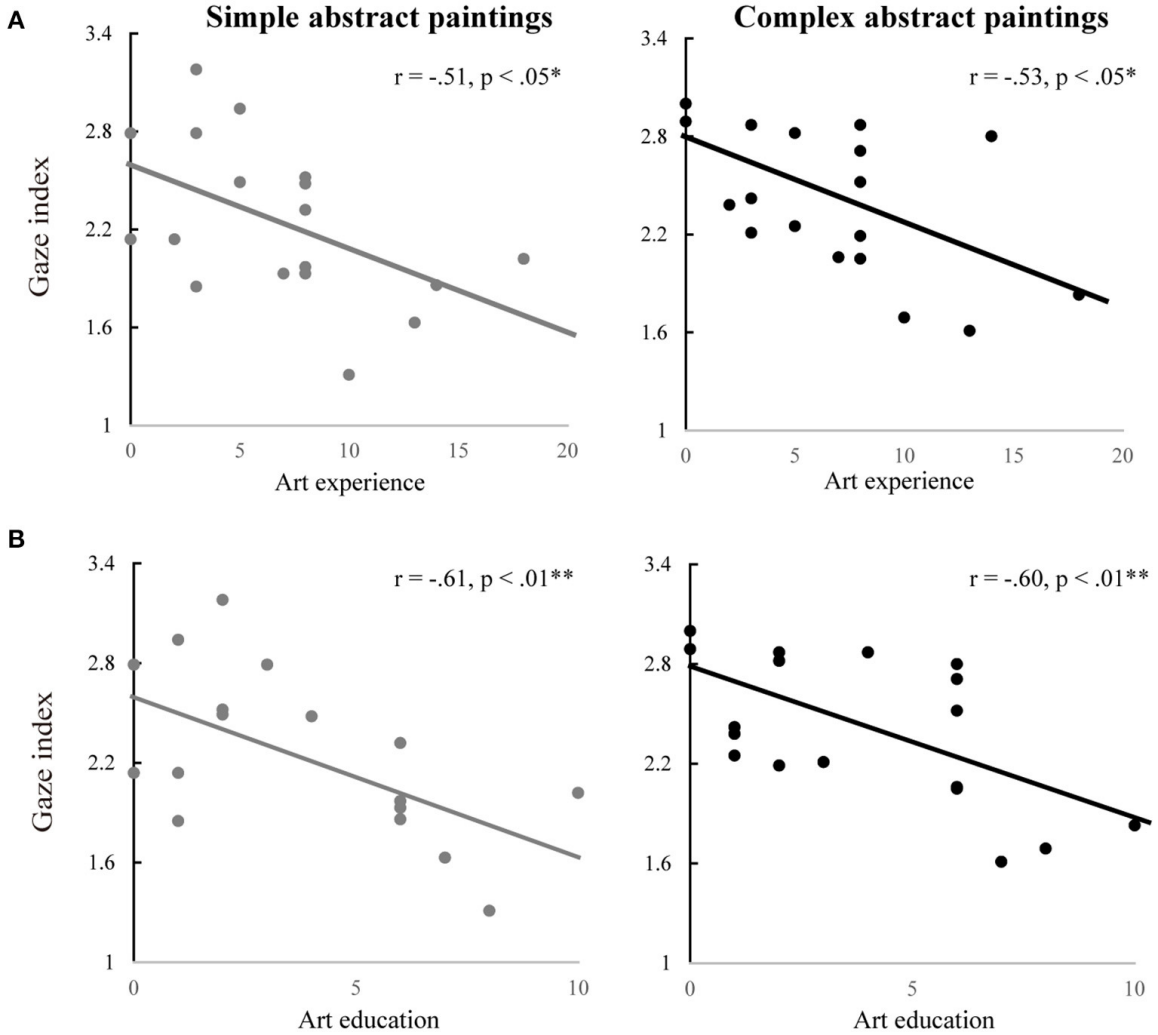
The ratio of fixation count and art experience in simple and complex abstract paintings. **(A)** Correlations between the gaze indices and art experience scores for the simple (left) and complex abstract paintings (right). **(B)** Correlations between the gaze index and art education for simple (left) and complex abstract paintings (right). *p < 0.05, **p < 0.01.

As shown in Figure 6A, there was a statistically significant negative correlation between the gaze indices and the art experience scores for simple, *r* = −0.51, *p* < 0.05, and complex abstract paintings, *r* = −0.53, *p* < 0.05. No statistically significant correlations were found for the other four conditions. Additional correlation analyses revealed that among the three subscales of art experience, education was correlated negatively with the gaze index for both simple, *r* = −0.61, *p* < 0.01, and complex abstract paintings, *r* = −0.60, *p* < 0.01, respectively (see Figure 6B).

Overall, these results indicate that participants who were less experienced in art, having less art education in particular, looked at a novel painting more frequently relatively to a familiar one in a greater degree when a pair of abstract paintings were presented than when a pair of representational paintings were presented.

## Discussion

In the present study we aimed to examine the extent to which aesthetic preference is driven by novelty and how familiarity-novelty interacts with preference of an artwork taking its content, visual complexity, and experience in art into consideration. By exploiting a sequential preference judgment paradigm, we found that familiarity-novelty preferences are affected by both stimulus and perceiver characteristics. Specifically, we demonstrated dissimilar patterns of familiarity-novelty preferences across different contents and different degrees of complexity in paintings. We also found that familiarity-novelty preference is mediated by the degree of art experience for abstract paintings in particular. Furthermore, the convergent results that were derived using behavioral and eye-tracking data permit us to draw more concrete conclusions about familiarity-novelty preferences for artworks, which have not been consistently drawn in previous studies.

Our results showed overall preference for novel compared to familiar paintings, which is in line with previous research showing the preference for novelty, challenges, and ambiguousness using behavioral (Jakesch and Leder, 2009; van de Cruys and Wagemans, 2011; Minissale, 2013; Muth et al., 2013, 2015; Belke et al., 2015; Muth and Carbon, 2016) and neuroscientific (Biederman and Vessel, 2006; Wittmann et al., 2007) measures. This supports the view that novelty is what observers seek for and root their aesthetic preference on (Imamoglu, 1974; Stang, 1974; Biederman and Vessel, 2006). It should be noted that in the present study, the concept of novelty cannot completely embrace the artistic values of novelty. Familiarity was also manipulated by repeated presentations. Needless to say, viewing an artwork in an experimental situation is not the same as viewing it in a real-world situation (Carbon, 2017, 2020). Nonetheless, the experimental paradigm we employed allowed us to clearly examine the effect of familiarity-novelty. Familiarity and novelty have not only been closely examined in the investigation into aesthetic appreciation (Leder, 2001; Cutting, 2003; Leder et al., 2004; Leder and Nadal, 2014), but they have been driving forces in the progress of art history as fundamental yet competing values (Martindale, 1990; Hekkert and van Wieringen, 1996). However, most existing models of familiarity-novelty preference did not shed light on the comprehensive effect of stimulus and perceiver properties on aesthetic preference. By revealing the interactive influence of painting content and complexity, these results provide evidence to clarify conflicting results on familiarity-novelty preference in previous studies. Moreover, the current study considering the mediating effect of art experience on familiarity-novelty preference is meaningful in that the individual characteristics of the viewer is critical in aesthetic appreciation and evaluation as well as the properties of the artworks (Feist and Brady, 2004). Results from the current study revealed that familiarity-novelty preference is derived from an interactive, multifaceted appreciation processes, which challenges an aesthetic universalism of many aesthetic appreciation models and makes our understanding of the important subject matter a step further and converging.

Our results demonstrated that novelty (or familiarity) preference relied on the depicted contents as well as complexity. Specifically, a novel painting was preferred over a familiar one when a pair of complex landscapes was presented. This result is in line with the previous studies showing novelty preference for natural scenes (Biederman and Vessel, 2006; Park et al., 2010; Liao et al., 2011). The complexity of the landscapes in the current study was represented by the quantity of the objects depicted as in the case of real-world scenes (Oliva et al., 2004). The complex landscapes with various objects, compared to the simple landscapes with the relatively limited number of objects, might have been perceived to our participants as more typical natural scenery eliciting greater activity in the scene processing regions of the brain (Chai et al., 2010). This might be a reason behind the more pronounced novelty preference in complex, relative to simple, landscapes. A novel painting was also preferred over a familiar one for a pair of abstract paintings, irrespective of the visual complexity of the pair. This is consistent with previous studies demonstrating a novelty preference for abstract patterns of images (Imamoglu, 1974; Stang, 1974, but see also Berlyne, 1970; Zajonc et al., 1972; Stang and O'Connell, 1974; Kruglanski et al., 1996).

The only exception to the overall novelty preference was the finding that a familiar painting was preferred to a novel one when a pair of simple portraits was presented. This result is in line with previous studies reporting general preference for familiar faces (Park et al., 2010; Liao et al., 2011). Given the perceptual and social significance of faces (Kanwisher et al., 1997; Allison et al., 2000), it is plausible that preference for a portrait reflects preference for a face depicted in the painting stemming from an increased familiarity and intimacy through repetitive exposure. Then, why is the familiarity preference for faces reflected only in the simple, but not in the complex portraits? This might be related to the way visual complexity was identified in our stimuli. As shown in Figure 1A, the degree of complexity in portraits was mainly dependent on the artistic style expressed by the number of colors, lines, and brushstrokes, rather than the number of objects. In other words, faces in the complex portraits were expressed with a variety of colors, unclear edges, and fine brushstrokes whereas faces in the simple portraits were straight representation of reality. Considering that aesthetic preference is influenced by the face contents in the naturalistic portraits more than expressive ones (Leder et al., 2013), it seems reasonable that familiarity preference was dominant for simple portraits in which the face contents were delivered in a similar way that we perceive faces in our daily lives.

This last point leads us to highlight the lack of the modulatory effect of complexity on novelty preference in abstract paintings that was present in representational paintings including portraits as well as landscapes. To discuss this result, it should be noted that the participants of the current study had no formal training in art or a degree in any art-related major. Previous studies have shown that viewers with limited experience in art tend to focus more on recognizable objects rather than on background and relations among elements (Nodine et al., 1993; Zangemeister et al., 1995; Vogt, 1999; Vogt and Magnussen, 2007; Cattaneo et al., 2015; Nadal et al., 2018). They also rely primarily on cognitive processes such as identifying depicted objects and understanding scenes (Cupchik, 1992; Winston and Cupchik, 1992; Nodine et al., 1993; Cela-Conde et al., 2004). In addition, their appraisal of a piece of art is guided mainly by semantic features (Parsons, 1987; Schmidt et al., 1989). Hence, our participants might have discovered nothing but meaningless brushstrokes when appreciating an abstract painting (Nadal et al., 2018) regardless of the complexity, which presumably led them to be satiated only after a few repeated views and to create bias in their preference toward a novel painting (Berlyne, 1970; Montoya et al., 2017).

Despite the overall novelty preference for abstract paintings, however, relative preference for pairs of abstract paintings continued to change over the course of trials. This was contrasted with representational paintings for which familiarity-novelty preference emerged early and tended to be maintained. This result implies yet another distinction between abstract and representational paintings, the distinction associated with the differential ways of aesthetic appraisals for the two categories of paintings. It has been shown that aesthetic appraisal of abstract paintings depends on internally presented information and knowledge (Jacobsen and Höfel, 2003). In contrast, aesthetic evaluation of representational paintings depends on externally presented information (Christoff and Gabrieli, 2000; Ridderinkhof et al., 2004; Cupchik et al., 2009) and is subserved by the left dorsolateral prefrontal cortex implicated in selecting, processing, and evaluating the information (Lengger et al., 2007; Cupchik et al., 2009). Additionally, the aforementioned characteristics of the viewer might have also come into play in the temporal fluctuation of the familiarity-novelty preferences in abstract paintings. Specifically, our participants with little experience and knowledge in art might have been unsure about what to look for in abstract paintings and how to evaluate them.

Our results based on both the behavioral and the gaze measures further buttress the influence of the viewer's experience in art on their familiarity-novelty preferences for abstract paintings. Among our participants with little experience in art, those who scored lower on the art experience questionnaire were more likely to prefer a novel painting over a repeated and familiar one for simple abstract paintings. Moreover, it is converged with the gaze results that they looked at a novel painting more frequently relative to a familiar one for both simple and complex abstract paintings. Specifically, these results were evident when art education was considered among the three subscales of art experience questionnaire. These correlations are consistent with the previous findings suggesting that art experts and non-experts tend to agree in their appraisals of representational artworks while they diverge on the evaluations of abstract artworks (Pihko et al., 2011; Leder et al., 2012, 2014; Mullennix and Robinet, 2018). Since abstract art is conceptually challenging to be appreciated and ambiguous in interpretation (Minissale, 2013; Leder and Nadal, 2014), the art-related knowledge acquired from art education can guide them to dissolve the ambiguities (Leder et al., 2004). Although our participants varied only in the relative degree of art experience and none of them were experts, it seems reasonable to infer that the individual variance was reflected in the differential effect of art experience, art education in particular, on familiarity-novelty preference for abstract paintings. People who are relatively more educated in art are less likely to be satiated with a repeated, familiar painting because they appreciate artworks based on their art-related knowledge and internal thoughts. Further studies are needed to test a group of art experts in comparison with a group of non-experts to examine the potential influence of expertise on familiarity-novelty preference for art.

The present study ensured that initial preferences were equally neutral across different painting content and complexity conditions. Painting stimuli were carefully chosen based on the results of a large-scale survey, and they were used to ensure that there was no statistically significant difference in preference across the six stimulus conditions. It is particularly important to control initial preference levels for several reasons. First, familiarity-novelty preference can be mediated by initial preference for stimuli. Studies have shown that repeated exposure to initially neutral stimuli enhances preference (Peeters, 1971; Kanouse and Hanson, 1972), whereas repeated exposure to initially negative stimuli hampers preference (Brickman et al., 1972). Second, current preference is affected by preference for an image that has been previously presented in a sequential preference judgment task (Cogan et al., 2013; Kondo et al., 2013; Tousignant and Bodner, 2014; Pegors et al., 2015; Chang et al., 2017; Khaw and Freedberg, 2018; Kim et al., 2019). If a combination of positive and negative paintings had been used in each stimulus condition, current preference may have also been influenced by prior preferences rather than solely by familiarity-novelty. Despite the potential issue of limited scope of the findings based on moderately familiar and moderately preferred paintings, the experimental consideration of the control has a greater advantage. By including only affectively neutral paintings and excluding the confounds of initial preferences for stimuli, the present study unveiled more detailed and concrete influences of painting content and complexity on familiarity-novelty preference. Further studies are needed to examine whether the results vary depending on whether the paintings are highly liked or disliked (Meskin et al., 2013; Belke et al., 2015).

In the present study, gaze was almost always directed toward novel over familiar painting images. This finding is consistent with the results of past studies, which have demonstrated support for a general tendency for gaze bias that favors novel images (Berlyne, 1958; Cantor and Cantor, 1966; Leckart, 1966; Faw and Nunnally, 1968; Glaholt and Reingold, 2009; Liao and Shimojo, 2012). At the same time, our gaze index unveiled the differential effects of painting content and complexity on familiarity-novelty preference echoing the behavioral results. Specifically, there was a larger gaze bias toward novelty for abstract than for representational paintings as well as for complex than for simple paintings. These results are in line with previous findings that people tend to fixate on (Plumhoff and Schirillo, 2009; Williams et al., 2018) and gaze for longer durations at (Holmes and Zanker, 2012) an aesthetically preferred paintings compared to less preferred ones. Our gaze index—i.e., the relative fixation counts between a novel and a repeated, familiar paintings—is an indirect indicator of aesthetic preference, which is associated with the novelty-driven saliency factor on gaze fixations. However, given the aforementioned results that the variances for initial familiarity were equal across the six painting conditions, the extent of stimulus novelty was controlled equally across the six conditions. Hence, the current results showing differential gaze indices between painting conditions cannot solely stem from the stimulus novelty. The source of variance is interpreted to be linked to aesthetic preference on the basis of previous findings demonstrating that the gaze fixation is a reliable predictor of preference (Shimojo et al., 2003; Simion and Shimojo, 2006, 2007; Glaholt and Reingold, 2009; Glaholt et al., 2009; Leder et al., 2010; Schotter et al., 2010; Mitsuda and Glaholt, 2014; Saito et al., 2017) and the behavioral preference results in the current study. The differential gaze biases toward representational and abstract paintings also imply the differences in the aesthetic appraisal of the two types of paintings. One may argue that complexity, not familiarity-novelty preference, was reflected in the gaze results showing a larger gaze bias toward novelty in abstract paintings and complex paintings, as it takes longer to process complex stimuli than simple stimuli (Berlyne, 1958; Faw and Nunnally, 1967, 1968; Lemond et al., 1974; Akai and Nakajima, 1989). However, this does not seem to be the case; the gaze ratio in simple abstract paintings was much higher than in simple portraits and simple landscapes although the mean complexity score of simple abstract paintings (*Z* = −0.54) was lower than that of simple portraits (*Z* = −0.35) or simple landscapes (*Z* = −0.36). Thus, gaze results cannot be solely accounted for the processing of complex paintings. This confirms that the gaze index is a valid predictor for familiarity-novelty preference supplementing the behavioral measure.

Yielding results that serve as significant empirical evidence, the present study entailed several methodological strengths. First, artistic paintings that were selected based on the results of a large-scale survey were used in the main experiment, thereby enhancing the generalizability of our results. Second, paintings were carefully selected to control those low-level visual elements including luminance and color, although colorfulness was not strictly controlled. Third, the use of a sequential preference judgment task allowed us to clearly examine the effect of familiarity-novelty and temporal changes in preference across the course of the trial. Therefore, the present results serve as an important piece of evidence on familiarity-novelty, which is one of the most critical factors that can elucidate the mysteries of aesthetic preference.

## Data Availability Statement

The raw data supporting the conclusions of this article will be made available by the authors, without undue reservation.

## Ethics Statement

The studies involving human participants were reviewed and approved by the Institutional Review Board of Korea University. The patients/participants provided their written informed consent to participate in this study.

## Author Contributions

C-YK conceived the research. C-YK, JS, and YK designed the experiment. JS conducted the experiment and drafted the manuscript. C-YK and JS analyzed the results. C-YK and YK provided critical revisions. All authors contributed to the article and approved the submitted version.

## Conflict of Interest

The authors declare that the research was conducted in the absence of any commercial or financial relationships that could be construed as a potential conflict of interest.

## Publisher's Note

All claims expressed in this article are solely those of the authors and do not necessarily represent those of their affiliated organizations, or those of the publisher, the editors and the reviewers. Any product that may be evaluated in this article, or claim that may be made by its manufacturer, is not guaranteed or endorsed by the publisher.

## Footnotes

^1^http://www.wikiart.org/
