# Supplementary material for: Familiarity and Novelty in Aesthetic Preference: The Effects of the Properties of the Artwork and the Beholder
Source: Front Psychol. 2021 Jul 23;12:694927. doi: 10.3389/fpsyg.2021.694927 (PMC8345014; doi:10.3389/fpsyg.2021.694927)
Supplement: Supplementary file 1 [file Data_Sheet_1.docx]

Supplementary Material

**LIST OF ARTWORKS**

The full list of paintings used in the main experiment.

**Simple portraits**

Berthe Morisot, Young Woman in a Hat, 1891

John Singer Sargent, Mrs.Charies Fairchild, 1887

Pierre-Auguste Renoir, Lady Smiling Portrait of Alphonsine Fournaises, 1875

Pierre-Auguste Renoir, Young Woman in Red Dress, 1892

Pierre-Auguste Renoir, Gabrielle in a Red Dress, 1908

Pierre-Auguste Renoir, Head of a Child, 1888

Berthe Morisot, Young Woman, 1871

Mary Cassatt, Portrait of a Young Woman in Green, 1898

Augustus Edwin John, Dylan Thomas, 1938

Pablo Picasso, Woman with cap, 1901

Pierre-Auguste Renoir, Jeanne Sisley, 1875

Georg Pauli, Untitled

Pierre-Auguste Renoir, Girl in a Red Hat, 1913

Paul Cezanne, Gustave Boyer in a Straw Hat, 1871

Pablo Picasso, Self-Portrait, 1901

William Merritt Chase,Portrait of a Woman

Pierre-Auguste Renoir, Aline Charigot, 1885

Pierre-Auguste Renoir, Stephane Mallarme, 1892

Camille Pissarro, Portrait of Jeanne, 1898

Mary Cassatt, Portrait of a Lady, 1890

Guy Rose, Miss C.

Pierre-Auguste Renoir, Jacques Eugene Spuller, 1871

Pierre-Auguste Renoir, Madame Henriot, 1874

Edgar Degas, Degas in a Green Jacket, 1856

Renoir, Head of a Woman, 1876

Edgar Degas,Man s Head

Pierre-Auguste Renoir, Woman Wearing a Hat, 1889

Pierre-Auguste Renoir, Gabrielle in a Red Blouse, 1913

Pierre-Auguste Renoir, Leonard Renoir, 1869

Pierre-Auguste Renoir, Paul Cezanne, 1880

Edouard Manet, Woman in Furs Portrait of Mery Laurent, 1882

Edgar Degas, Portrait of Josephine Gaujelin, 1867

John Singer Sargent, Mrs.William Russell Cooke, 1895

John Singer Sargent, Portrait of Louis Alexander Fagan, 1893

Tom Roberts, Edward Ogilvie, 1895

Valentin Serov, Portrait of actress M.Ya.Van-Zandt, in a marriage Cherinova, 1886

Pierre-Auguste Renoir, Self-Portrait, 1899

Augustus John, Mrs Randolph Schwabe, 1917

Edgar Degas, The Savoy Girl, 1873

Max Liebermann, Portrait of the publisher Bruno Cassirer, 1921

Viktor Vasnetsov, Portrait of Victor Goshkevich, 1887

Robin John, Robin John, 1916

Mary Cassatt, Portrait of Mrs William Harrison, 1890

Henri Matisse, Portrait of Michael Stein, 1916

Paul Cezanne, Portrait of Louis Guillaume, 1880

Edouar Manet, Portrait of a Lady with a Black Fichu, 1878

Edouar Manet, Portrait-of-omposer Emmanual Chabrier, 1880

Max Liebermann, Portrait of Professor Dr. Carl Bernstein, 1892

**Complex portraits**

Pierre-Auguste Renoir, Portrait of Henri Lerolle, 1895

Mary Cassatt, Portrait of Master St.Pierre, 1892

Mary Cassatt, Marie Therese Gailiard, 1894

Nikolay Bogdanov-Belsky, Portrait of Actor of Moscow Art Theatre

Mary Cassatt, Young Woman in Green Outdoors in the Sun, 1914

Pierre-Auguste Renoir, First Portrait of Madame Georges Charpeitier, 1877

Pierre-Auguste Renoir, Algerian Woman, 1881

Edouard Manet, The painter of Animals, 1882

Camille Pissarro, Portrait of Madame Pissarro, 1883

Pierre-Auguste Renoir, Wilhelm Muhfeld, 1910

Berthe Morisot, Young Woman in Mauve, 1880

Vincent van Gogh, Portrait of Madame Trabuc, 1889

Pierre-Auguste Renoir, Portrait of a Young Woman, 1876

John Singer Sargent, Carmela Bertagna, 1879

Pierre-Auguste Renoir, Portrait of Eugene Pierre Lestringuez, 1878

Mary Cassatt, Head of a Young Girl, 1876

Pierre-Auguste Renoir, Portrait of a Young Girl, 1879

John Singer Sargent, Portrait of Jeanne Kleffer, 1879

Pierre-Auguste Renoir, Eugene Murer, 1877

Joaquín Sorolla, Portrait of a Caballero, 1884

Mary Cassatt, Portrait of Charies Dikran KeleKian, 1910

Gustave Caillebotte, Bust Portrait of Eugene Lamy, 1888

Berthe Morisot, Young Woman in a Blue Blouse, 1891

Augustus John, Richard Hughes, 1937

Henri de Toulouse-Lautrec, Man’s Head, 1883

Vincent van Gogh, Portrait of Trabuc and Attendant at Saint-Paul Hospital, 1889

Berthe Morisot, Woman with a Fan, 1876

John Singer Sargent, Francisco Bernareggi, 1908

Paul Cezanne, Portrait of Victor Chocquet, 1877

Camille Pissarro, Paul Emile Pissarro, 1890

Paul Cezanne, Portrait of Joachim, 1896

Mary Cassatt, Susan in a Toque Trimmed with Two Roses, 1881

Joaquín Sorolla, Portrait of a Caballero, 1884

Berthe Morisot, Jeannie Gobillard, 1894

John Singer Sargent, Mrs.William Shakespeare Louise Weiland, 1896

Pierre-Auguste Renoir, Portrait of Nini Lopez, 1876

John Singer Sargent, Portrait of Jacques Emile Blanche, 1886

Pierre-Auguste Renoir, Madame Claude Monet, 1872

Berthe Morisot, Young Woman on a Couch, 1885

Edgar Degas, Mademoiselle Malo, 1877

Pierre-Auguste Renoir, Portrait of the Countess of Pourtalès, 1877

Camille Pissarro, Portrait of Madame Felicie Vellay Estruc, 1874

Merritt Chase, portrait-of-elizabeth-betsy-fisher, 1899

Pierre-Auguste Renoir, Self-Portrait, 1875

Pierre-Auguste Renoir, The Artist a Mother, 1860

Vincent van Gogh, Portrait of Woman in Blue, 1885

Pierre-Auguste Renoir, Richard Wagner, 1882

Vincent van Gogh, Portrait of a Man with a Skull Cap, 1887

**Simple landscapes**

Childe Hassam, golden afternoon, 1908

Pierre-Auguste Renoir, Strong Wind Gust of Wind 1872

Claude Monet, The Pave de Chailly, 1865

Claude Monet, The Arm of the Siene at Jeufosse, Afternoon, 1884

Robert Henri, Pequot Light House, Connecticut Coast, 1902

Gustave Caillebotte, Promenade at Argenteuil, 1883

Vincent van Gogh, Wheat Field, 1888

Gustave Calibotte, The Seine and the Railroad Bridge at Argenteuil, 1886

Paul Gauguin, Coastal Landscape, 1886

Paul Cezanne, Factories Near Mont de Cengle, 1870

Pierre-Auguste Renoir, The Bay

Joaquín Sorolla, View of Las Pedrizas from El Pardo

Childe Hassam, Isle of Shoals, 1912

Gustave Caillebotte, Woods at La Grange, 1879

Paul Cezanne, Chestnut Trees at the Jas de Bouffan, 1887

Childe Hassam, Cliff Rock – Appledore, 1903

Vincent van Gogh, Farmhouse in a Wheat Field, 1888

William Merritt Chase, Brooklyn Landscape, 1886

Eugene Boudin, Etaples La Canache High Tide, 1890

Robert Henri, Ship in the Bay, 1903

Camille Pissarro, Landscape near Pontoise, 1880

Theodore Clement Steele, Okemo Mountain, Ludlow, Vermont, 1887

Gustave Caillebotte, Garden at Yerres, 1876

William Merritt Chase, Brooklyn Navy Yard, 1887

Wilard Metcalf, Pond Giverny, 1884

Eugene Boudin, The Berck Dunes, 1886

Armand Guillaumin, Cottages in a Landscape, 1896

Paul Cezanne, Chestnut Trees and Farmstead of Jas de Bouffin, 1876

Camille Pissarro, Village Church, 1868

Paul Gauguin, Farm in Brittany, 1894

Vincent van Gogh, Farmhouse in Loosduinen near The Hague at Twilight, 1883

Pierre-Auguste Renoir, Landscape with Fence, 1910

Alfred Sisley, Snow on the Road, Louveciennes, 1874

Childe Hassam, Mount Hood, 1908

William Merritt Chase, Gravesend Bay (aka The Lower Bay), 1889

Alfred Sisley, Avenue of Chestnut Trees near La Celle Saint Cloud, 1865

Pierre-Auguste Renoir, Banks of a River, 1896

Pierre-Auguste Renoir, The Banks of the River

William Merritt Chase, Repair Docks Gowanus Pier, 1888

John Singer Sargent, A Mountain Stream Tyrol, 1914

Alfred Sisley, The Seine at Bougival in Winter, 1872

Berthe Morisot, Thatched Cottage in Normandy, 1865

Joaquín Sorolla, Storm over Penalara, Segovia, 1906

Gustave Caillebotte, Cliff at Villers sur Me, 1880

Gustave Caillebotte, Meadow Bridge at Argenteuil, 1886

Claude Monet, Sunset on the Seine in Winter, 1880

Armand Guillaumin, Valley in Pontgibaud, 1890

Paul Gauguin, The Market Gardens of Vaugirard, 1879

**Complex landscapes**

Childe Hassam, Village Scene, 1885

Armand Guillaumin, Arpes la pluie, 1885

Claude Monet, The Cliffs of Le Bouille, 1884

Gustave Caillebotte, Boats on the Seine at Argenteuil, 1890

Gustave Caillebotte, The Seine at Epinay, 1888

Camille Pissarro, The Village of Eragny, 1885

Camille Pissarro, Sunset at Eragny

Claude Monet, Road of La Roche Guyon, 1880

Paul Gauguin, Le Port de Dieppe, 1885

Claude Monet, Strada Romada in Bordighera, 1884

Gustave Caillebotte, Landscape at Argenteuil, 1889

Gustave Caillebotte, Laundry Drying, 1892

Paul Gauguin, Garden under Snow, 1879

Pierre-Auguste Renoir, Rocky Craggs at l'Estaque, 1882

Ernest Lawson, Washington Bridge, New York City

Camille Pissarro, Autunm in Eragny, 1899

Pierre-Auguste Renoir, The Rose Garden at Wargemont, 1879

Alfred Sisley, Barges on the Loing at Saint Mammès, 1885

Gustave Caillebotte, The Pontoon at Argenteuil 1887

Stanislas Victor-Édouard Lepine, The Island of La Grande Jatte in Summer

Alfred Sisley, The Station at Sevres, 1879

John Singer Sargent, Oranges at Corfu, 1909

Camille Pissarro, Field at Eragny, 1885

Paul Cezanne, The Bay of l’Estaque and Saint-Henri, 1879

Paul Cezanne, In the Oise Valley, 1874

Gustave Caillebotte, Garten in Trouville, 1882

Camille Pissarro, Le Valhermeil near Pontoise, 1880

Pierre-Auguste Renoir, Cape Saint Jean, 1910

Pierre-Auguste Renoir, Rocks at I’Estaque, 1882

John Singer Sargent, The Moraine, 1908

Claude Monet, Poplars at Giverny, 1887

Childe Hassam, Spring, Navesink Highlands, 1908

Alfred Sisley, Moret-sur-Loing, 1888

Theodore Clement Steele, Mysterious, 1895

Alfred Sisley, Moret, The Banks of the River Loing, 1877

Gustave Caillebotte, The Petit Bras of the Seine, 1888

Eugene Boudin, Oiseme Landscape near Chartres, 1893

Pierre-Auguste Renoir, View at Guernsey, 1883

Pierre-Auguste Renoir, Landscape, 1900

Childe Hassam, Road to the Land of Nod, 1910

Ernest Lawson, Approaching Storm, 1919-1920

Ernest Lawson, Spring, 1913

Armand Guillaumin, Saint-Julien-des-Chazes, 1895

Claude Monet, Apple Trees on the Chantemesle Hill, 1878

Paul Cezanne, Mill on the Couleuvre at Pontoise, 1881

Ernest Lawson, New England Birches

Paul Gauguin, Banks of the Oise, 1881

John Singer Sargent, Home Fields, 1885

**Simple abstract paintings**

Paul Klee, Harbour with Sailing Ships, 1937

Kazimir Malevich, Suprematic Painting, 1916

Fernand Leger, The Shapes on White Background

Auguste Herbin, Blanc, 1947

Giacomo Balla, Iridescent Compenetration, 1913

Fernand Léger, Mural, 1953

Willi Baumeister, Ideogram, 1938

Mark Rothko, Yellow, Cherry, Orange, 1947

Robert Delaunay, The Joy of Life, 1930

Jean Arp, Collage with Square Arranged According to the Laws of Chance, 1917

Mark Rothko, No.21, 1949

Francis Picabia, Abstract Composition, 1937

Arthur Dove, Sails, 1912

Mark Rothko, Multiform, 1948

Fernand Léger, Composition on an orange background, 1932

Willi Baumeister, Bluxao, 1955

Paul Klee, Signs in Yellow, 1937

Paul Klee, Rock Chamber, 1929

Paul Klee, Halme, 1940

Ben Nicholson, Painting - still life, 1936

Sam Francis, Untitled (SFP53-31), 1953

Willi Baumeister, Aru with Yellow, 1955

Vasile Dobrian, Untitled, 1997

Auguste Herbin, Untitled, 1931

Lawren Harris, Abstract

Kazimir Malevich, Suprematism and with Eight Red Rectangles, 1915

Giacomo Balla, Line of Speed, 1913

Hans Hofmann, Black Diamond, 1961

Paul Klee, Park of Idols, 1938

Fernand Léger, Not identified

Mark Rothko, No.3/No.13 (Magenta, Black, Green on Orange), 1949

Arthur Dove, Foghorns, 1929

Kazimir Malevich, Composition, 1932

Fernand Léger, Mural, 1926

Jean Arp, Geometric Forms, 1914

Hans Hofmann, Above Deep Waters, 1959

Frantisek Kupka, Vertical Plains Blue and Red, 1913

Jean Arp, Constellations, 1938

Vasile Dobrian, On Both Sides Our Worries No Longer Exist!, 1969

Hans Hofmann, Silent Night, 1964

Ben Nicholson, 1924 (first abstract painting, Chelsea), 1924

Vasile Dobrian, Composition, 1989

Auguste Herbin, Untitled, 1959

Mark Rothko, Untitled, 1959

Hans Hofmann, Morning Mist, 1958

Robert Delaunay, Formes circulaires lune no.1, 1913

Willem de Kooning, Door to the River, 1960

Willem de Kooning, The Wave, 1942

**Complex abstract paintings**

Stuart Davis, Hot Still Scape for Six Colors - 7th Avenue Style, 1940

Paul Klee, Hamamet, 1914

Francis Picabia, Udnie, Young American Girl, 1913

Fernand Léger, The Disc, 1918

Frantisek Kupka, Organization of Graphic Motifs, 1912

Ben Nicholson, Poisonous Yellow 5 December, 1949

Willi Baumeister, Phantom and Yellow Table, 1952

Ben Nicholson, August 1956, 1956

Marsden Hartley, Abstraction, 1914

Fernand Léger, Composition, 1919

Robert Delaunay, Rhythm, 1939

Marsden Hartley, Painting, Number 5, 1915

Paul Klee, Tropical Garden, 1919

Willem de Kooning, Secretary, 1948

Paul Klee, A Kind of Cat, 1937

Werner Drewes, Composition 67 in Blue, 1934

Willi Baumeister, Machine Man with Spiral Turn, 1930

Umberto Boccioni, Elasticity, 1912

Robert Delaunay, Circular Forms, 1930

Willi Baumeister, Three Stepped Figures, 1920

Wassily Kandinsky, Composition 4, 1911

Hans Hofmann, The Lark, 1960

Lawren Harris, Nature Rhythms, 1950

Piet Mondrian, Not identified, 1913

Paul Klee, Transparent in Perspective Grooved, 1921

Giacomo Balla, Planet Mercury passing in front of the Sun, 1914

Fernand Léger, July 14, 1914

Jackson Pollock, The Key, 1946

Wassily Kandinsky, Black Spot, 1912

Wassily Kandinsky, 304, 1910

Fernand Léger, Composition, 1920

Fernand Léger, Contrasts of Forms, 1913

Frantisek Kupka, Disks of Newton, Study for Fugue in Two Colors, 1911

Lawren Harris, Untitled, 1951

Pablo Picasso, The Bird Cage, 1944

Robert Delaunay, Simultaneous Windows, 1912

Robert Delaunay, Circular forms. Sun and Moon, 1912/1931

Giacomo Balla, Pessimism and Optimism, 1923

Adnan Coker, Unknown Title

Wassily Kandinsky, Improvisation.Deluge, 1913

Frantisek Kupka, Two Grays II, 1928

Paul Klee, Highway and Byways, 1929

Fernand Léger, The level Crossing, 1912

Ben Nicholson, Feb 2-54, 1954

Wassily Kandinsky, Improvisation 26(Rowing), 1912

Frantisek Kupka, Positioning of Mobile Graphic Elements, 1913

Hans Hofmann, The Prey, 1956

Giacomo Balla, Iridescent Interpenetration No.5 - Eucalyptus, 1914
